# Fabrication of Antibacterial Metal Surfaces Using Magnetron-Sputtering Method

**DOI:** 10.3390/ma14237301

**Published:** 2021-11-29

**Authors:** Agata Markowska-Szczupak, Oliwia Paszkiewicz, Beata Michalkiewicz, Adrianna Kamińska, Rafał Jan Wróbel

**Affiliations:** 1 Department of Chemical and Process Engineering, Faculty of Chemical Technology and Engineering, West Pomeranian University of Technology in Szczecin, Piastów Ave. 42, 71-065 Szczecin, Poland; po43902@zut.edu.pl; 2 Department of Catalytic and Sorbent Materials Engineering, Faculty of Chemical Technology and Engineering, West Pomeranian University of Technology in Szczecin, Piastów Ave. 42, 71-065 Szczecin, Poland; Beata.Michalkiewicz@zut.edu.pl (B.M.); ka47829@zut.edu.pl (A.K.); rafal.wrobel@zut.edu.pl (R.J.W.)

**Keywords:** antibacterial, copper-gold-silver, magnetron sputtering

## Abstract

One-hundred-nanometer films consisting of silver, copper, and gold nanocrystallites were prepared, and their antibacterial properties were quantitatively measured. The magnetron-sputtering method was used for the preparation of the metallic films over the glass plate. Single- and double-layer films were manufactured. The films were thoroughly characterized with the XRD, SEM, EDS, and XPS methods. The antibacterial activity of the samples was investigated. Gram-negative *Escherichia coli*, strain K12 ATCC 25922 (*E. coli*), and Gram-positive *Staphylococcus epidermidis,* ATCC 49461 (*S. epidermidis*), were used in the microbial tests. The crystallite size was about 30 nm in the cases of silver and gold and a few nanometers in the case of copper. Significant oxidation of the copper films was proven. The antibacterial efficacy of the tested samples followed the order: Ag/Cu > Au/Cu > Cu. It was concluded that such metallic surfaces may be applied as contact-killing materials for a more effective fight against bacteria and viruses.

## 1. Introduction

The global COVID-19 pandemic has raised interest towards the development of advanced antiviral and antibacterial surfaces, films, coatings, and materials. Many new research reports in this field are being added every day to the literature. Besides the direct route (from human to human), the indirect means of SARS-CoV-2 transmission (by contact with surfaces in the immediate environment or with objects used by the infected person) is also possible [1,2]. The antimicrobial coating market reached a value of USD 3.3 billion in 2020 and is projected to grow to over USD 5.6 billion by 2025, at an annual growth rate of 10.7% from 2020 to 2025 [3]. Nanomaterials and nanoparticles play an important role as antiviral and antibacterial agents that can kill or inhibit the growth of viruses, bacteria, and fungi. [4,5]. Since ancient times, silver, gold, and copper have been used to limit antimicrobial activity. Ag, Cu, and Au nanoparticles have also been used as germ-destroying agents [6]. They were investigated before the ongoing pandemic, but over the past two years there has been growing interest in this topic [7,8,9]. The scientific rationales for addressing this issue are: (i) metals can efficiently prevent virus and bacteria transmission [10]; (ii) bacterial/SARS-CoV-2 coinfections and secondary bacterial “super-infection” after SARS-CoV-2 play a significant role during COVID-19 infection [11,12]; (iii) the SARS-CoV-2 pandemic is superimposed on the ongoing pandemic of antibiotic-resistant bacteria (ARB); (iv) the common use of sanitizers and antibacterial agents as personal measures can enhance the transmission of antibiotic-resistance genes (ARG) [13]; and (v) microbial resistance to metal NPs is almost impossible [6,14,15].

Many coating techniques have been applied to obtain an antimicrobial surface. Because the production of metal coatings utilizes high-temperature techniques, it is very difficult to deposit thin films of metals on temperature-sensitive surfaces such polymers, glass, or textiles. The magnetron-sputtering process makes it possible to put coatings on temperature-sensitive objects. Magnetron sputtering is a well-known technique applied to prepare metallic nitrides, carbides, oxides, or metallic coatings [16,17]. Unfortunately, this process has seldom been applied for antiviral and antibacterial film production. Films of anatase, rutile, and their mixtures have been produced using a magnetron-sputtering technique [18]. The greatest antibacterial activity was achieved with the mixture film. Copper has been deposited on a thallium surface using magnetron sputtering. Ti6Al4V disks were coated with Ag, Cu, and Ag-Cu thin films using high-power impulse magnetron-sputtering in order to obtain antibacterial properties [19]. The Ag-Cu coatings were the most efficient in achieving the inactivation of *Escherichia coli* and *Pseudomonas aeruginosa*. The activity depended on the Ag/Au ratio. The antimicrobial properties of thin-film coatings consisting of Ti-Ag, Ti-Au, Ti-Cu, Ti, Ag, Au, and Cu on TiAlV alloy, Si, and SiO_2_ glass have been examined [20]. The strongest antifungal and antibacterial activity was shown for Ti-Cu. Zr-Cu-Ag thin films deposed on glass and silicon by magnetron sputtering for antibacterial applications has also been described. Their antibacterial effect against *Staphylococcus aureus* was proved [21]. 

Accordingly, in this study, Cu, Ag, and Au were deposited on glass plates using the magnetron-sputtering method and investigated for activity against the Gram-negative bacteria *Escherichia coli* and the Gram-positive *Staphylococcus epidermidis*, in order to clarify their germ-destroying properties.

## 2. Materials and Methods

### 2.1. Magnetron Sputtering

The coatings were obtained by magnetron-sputtering method with the use of a magnetron coater (Q150T; Quorum Technologies, Sacramento, CA, USA) equipped with a turbomolecular pump. The following conditions were used: deposition temperature 20 °C, base pressure 1.0 × 10^−2^ mbar, Ar flow 50 cm^3^/min, sputtering power 40 W, DC mode. The thickness of the deposited metallic film was monitored with the use of a quartz balance, which enables 0.1 nm measurement resolution. A quartz balance sensor was positioned on the same level as the substrates prepared for metal covering. The substrates were placed on the rotary stage 10 cm below the metal target. Ag, Au, and Cu targets with a diameter of 57 mm and purity of 99.99% were used as targets. The process was performed at the pressure of 2 mbar with the application of argon plasma. Thin nanofilms (100 nm) were deposited on glass plates of 1.0 cm × 1.0 cm. An ion current of 100 mA was applied. The following samples were obtained: Cu; Au; Ag; Cu/Ag; Cu/Au; Ag/Cu; Au/Cu; Ag/Au; and Au/Ag. In terms of notation, Ag/Cu, for example, denotes that the sequence of layers was in the following order: glass/50 nm Ag/50 nm Cu, i.e., the Cu layer was on top.

### 2.2. X-ray Photoelectron Spectroscopy (XPS)

X-ray photoelectron spectroscopy measurements were performed in a commercial multipurpose (XPS, AES, LEED, UPS) ultra-high vacuum (UHV) surface-analysis system (PREVAC). The base pressure attainable in the system was low, in the 10^−10^ mbar range. The UHV system consisted of preparation and analysis chambers. The analysis chamber was equipped with a kinetic electron energy analyzer (Spectrometer XPS, SES-2002, Scienta Scientific AB, Uppsala, Sweden, 2002) and nonmonochromatic X-ray photoelectron spectroscopy (XPS). The calibration of the spectrometer was performed using Ag 3d_5/2_ transition. Samples in the form of plates covered with metal were mounted on a sample holder. The metal surface deposited on the glass was connected to the ground to avoid charging effects. The samples were thoroughly degassed under vacuum for 3 h prior to measurement. The vacuum during XPS measurements was in the low 10^−9^ mbar range. The X-ray photoelectron spectroscopy was performed using Mg K_α_ (hν = 1253.7 eV) radiation.

### 2.3. X-ray Diffraction (XRD)

The measurements for thin films were performed with the use of a multipurpose diffractometer (Empyrean; PANalytical, Malvern, UK) equipped with an X-ray tube with copper anode (Cu λ_Kα_ = 0.15406 nm). The measurement range was 30–120 degrees in 2θ scale with a 0.026 degree step. The X’Pert HighScore software (PDF-4+ 2020 International Centre for Diffraction Data databases) was used to identify phases and evaluate data. All metals exhibited a face-centered cubic crystallographic system. For metals Cu, Ag, and Au, the ICDD numbers 04-001-3178, 04-003-5319, and 01-089-3697 were assigned, respectively.

### 2.4. Scanning Electron Microscopy (SEM) Coupled with Energy Dispersive Elemental Analysis (EDX)

The measurements for thin films were performed with the use of a high-resolution scanning electron microscope (SU8020 Ultra-High Resolution Field Emission Scanning Electron Microscope; Hitachi Ltd., Tokyo, Japan, 2012) equipped with a cold field electron emitter. The setup was screened against changes of magnetic field enabling high-quality ultra-high magnification of 200,000 times. The samples in the form of glass plates covered with thin metallic film were mounted over the sample holder with conductive carbon tape. Additionally, in order to avoid charging effects, the metal surface was connected to the ground, also with conductive carbon tape. A 5 kV accelerating voltage was applied for the SEM images. 

The elemental composition of the thin metallic surfaces was characterized with an analyzer of X-ray photons emitted from the sample during electron bombardment. The Energy Dispersive Spectroscopy system (Thermo Scientific Ultra Dry, Pittsburgh, PA, USA) was applied. The samples were excited with 20 keV electrons during elemental analysis. 

### 2.5. Microbial Tests

Bacterial strains and growth conditions. Gram-negative *Escherichia coli*, strain K12 ATCC 25922 (*E. coli*), and Gram-positive *Staphylococcus epidermidis* ATCC 49461 (*S. epidermidis*) were used as reference strains (ATCC—American Type Culture Collection). *E. coli* was grown in Enrichment Broth (BIOCORP Sp. z.o.o., PL) and *S. epidermidis* in Brain Heart Infusion Broth BHI (BIOCORP Sp. z.o.o., PL). All strains were incubated at 37 °C for 24 h, and activated by two successive transfers. The overnight cultures of *E. coli* were transferred to solutions prepared from reagent-grade chemicals (Chemland, PL): sterile saline buffer (0.85% NaCl) for *E. coli* and TBS buffer (50 mM Tris-Cl, 150 mM NaCl pH 7.5) for *S. epidermidis*. Cultures were diluted with appropriate buffers until the final concentration of bacteria was in the range of approx. 1.5–3.0 × 10^6^. All materials were tested according to the standard test method for determining the antimicrobial activity of antimicrobial agents under dynamic contact conditions, with our own modifications [22]. Glass plates of the size 1.0 cm × 1.0 cm were covered by various magnetron coatings and controls were sterilized under a UV-C lamp for 15 min. Treated and untreated specimens were placed into a sterile buffer in 250 mL screw-cap Erlenmeyer flasks. Then, 50 ± 0.5 mL of the working dilution of prepared bacterial inoculum were added to the flask. The series of flasks on the wrist-action shaker at 37 °C were shaken at maximum stroke for 3 h. An amount of 0.5 mL was taken as a sample after 1, 2, and 3 h. Bacterial concentration at the “0” time and during experiment was measured by serial dilutions and standard plate-count techniques in triplicate. Plate Count Agar (BIOCORP Sp. z.o.o., PL) was used for *E. coli* and Brain Heart Infusion Agar (BIOCORP Sp. z.o.o., PL) for *S. epidermidis*. All Petri dishes were incubated at 37 ± 2 °C for 24 h. The visible bacteria colonies were counted and were reported as colony-forming unit per millilitre (CFU/mL). The logarithmic (log) bacteria reduction from initial “0” time was calculated.

## 3. Results

**Figure 1:**
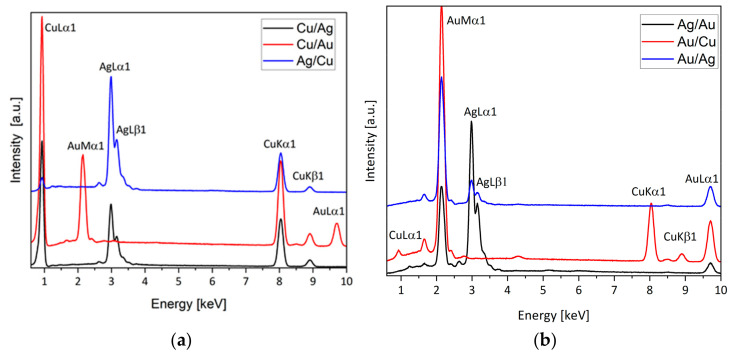
EDX spectra of double coatings: (**a**) Cu/Ag, Cu/Au, and Ag/Cu; (**b**) Ag/Au.

The single and double coatings of copper (Cu), silver (Ag), and gold (Au) obtained by the magnetron-sputtering method were tested. On the basis of the EDX measurements (Figure 1), the metal content in the double coatings was estimated (Table 1).

**Figure 2:**
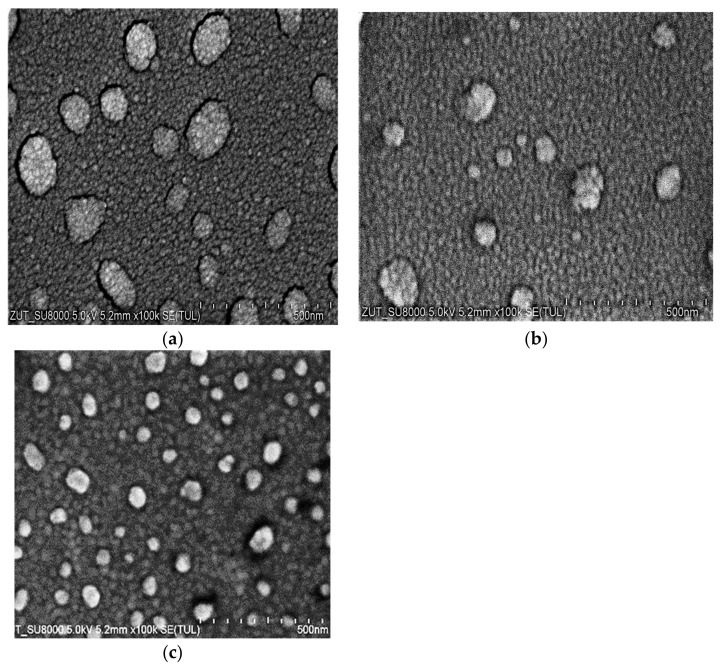
SEM images of the coatings at magnification of 100,000: (**a**) Cu; (**b**) Ag; (**c**) Au.

The microstructure of the coatings was examined using SEM and is presented in Figure 2. The SEM images showed that the morphology of the coatings was homogenous, and they had a fine-grained nanometric structure. The films consisted of small grains, and there were also clusters consisting of similar small grains on the films. On the basis of detailed SEM investigations, the average sizes of the Au and Ag grains were found to be roughly 30 nm. The size of the Cu grains could not be estimated; they were too small, and it was visible at a very high magnification that their size was considerably smaller than 30 nm. We were able to estimate the size of the clusters on the grain films. The size of the particles on the films was as follows: Cu, 50–200 nm; Ag, 25–150 nm; Au, 25–90 nm.

**Figure 3:**
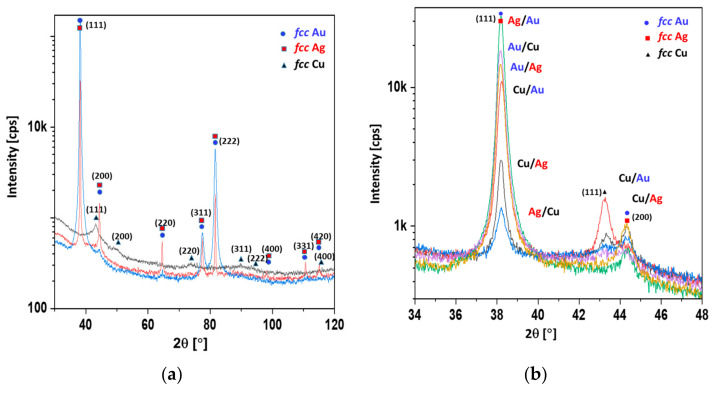
(**a**) XRD patterns of 100 nm Ag, Au, and Cu single coatings; (**b**) XRD patterns of double coatings.

In Table 2, the mean crystallite sizes calculated according to the Scherrer equation are presented. The crystallite sizes of gold, copper, and silver were evaluated from reflections at about 38, 43, and 44 degrees in 2θ scale (c.f. Figure 3). In the case of the Ag/Au and the Au/Ag coatings, the reflection of the gold overlapped with the reflection originating from the silver. Therefore, in the fifth column of Table 2, a value for the mixture of Au/Ag crystallites was given. 

It is evident from Table 2 that the copper crystallites could be as much as one order of magnitude smaller than those of the silver and gold. Moreover, the copper layer deposited under the silver or gold layer exhibited a larger mean crystallite size. The top layer of copper was identified as consisting of smaller copper crystallites, due to oxidation. This could indicate a core–shell model of oxidation, i.e., a small copper crystallite core enveloped by an amorphous copper oxide layer (shell). The copper layer located underneath gold or silver can be protected from oxidation, and thus exhibits larger crystallites. In Figure 3, the XRD spectra of 100 nm overlayers of Cu, Ag, Au, and double coatings are presented.

**Figure 4:**
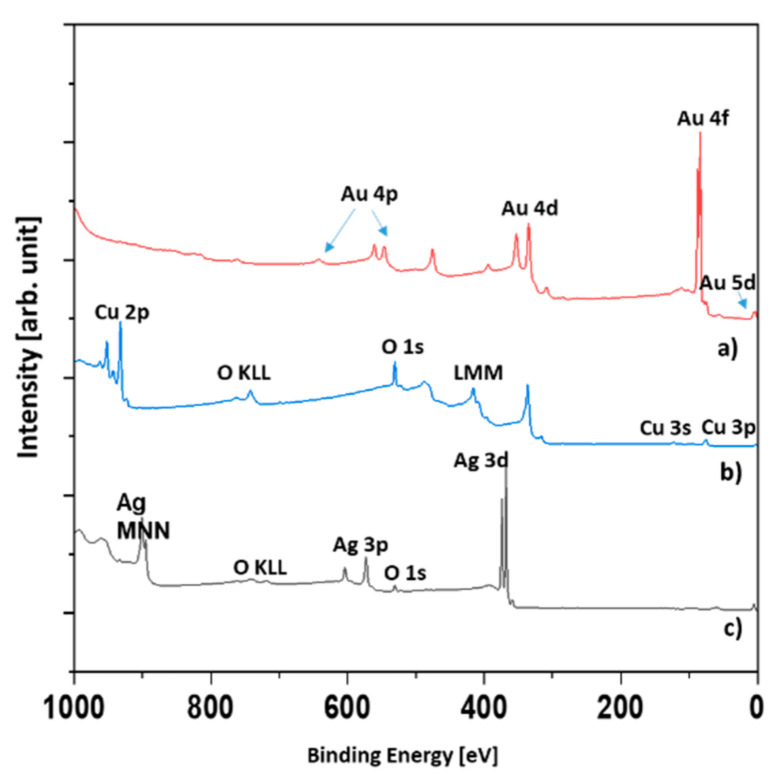
XPS survey spectra: Au, Ag, and Ag/Cu.

It was shown that the width at half the maximum reflection was inversely proportional to the mean crystallite size (Figure 3a). The crystallite sizes observed with the SEM method (Figure 2) were comparable to those obtained with the Scherrer method (Table 2). It is worth noting that the amorphous substance did not contribute to the reflection, but did contribute to the level of background. The highest backgrounds were exhibited by the diffractograms of copper and silver, indicating the significant content of the amorphous phase (Figure 3a). The reflex at about 38° in 2θ scale corresponded to silver and gold, whereas the reflexes at about 43° and 44° corresponded to copper and silver, respectively. It was shown that the strongest reflexes of copper corresponded to the Cu/Au and Cu/Ag samples and were far fainter for the Au/Cu and Ag/Cu samples. However, in the Cu/Au and Cu/Ag samples, the Au and Ag overlayers protected the copper from oxidation, resulting in a stronger copper signal, despite the attenuation effect (c.f. Figure 3b). With this assumption, the observed phenomenon was clear (Figure 3b). In Figure 4, the XPS spectra of the Au, Ag, and Ag/Cu samples are presented.

The reason why only three samples were investigated was that in the XPS technique, the signal originates roughly from 1 nm depth. Therefore, it was highly unlikely that signals other than those originating from the topmost layer could be observed. We tested only the Ag/Cu sample. As was expected, there was no silver signal. It was assumed that the XPS spectra of the Cu and Ag/Cu samples were the same. 

**Figure 5:**
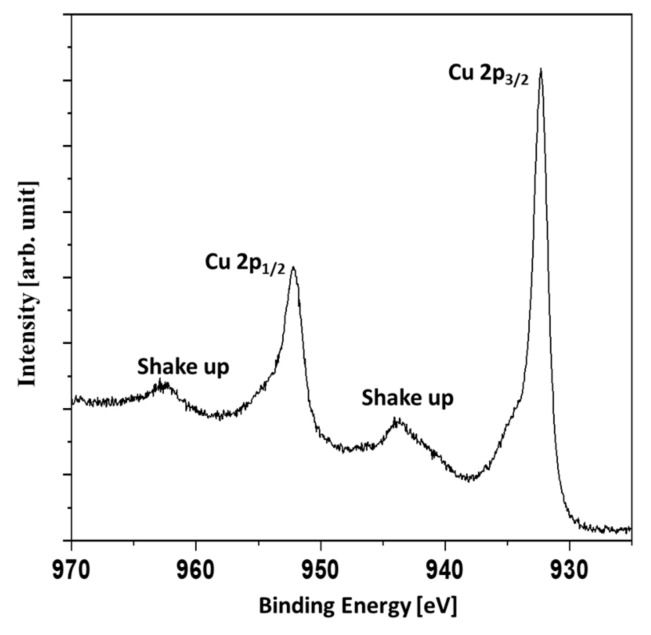
XPS Cu 2p high-resolution region for Ag/Cu sample.

The evaluation of the survey spectra enabled the quantitative determination of the surface composition (Table 3). In the case of the Ag and Ag/Cu samples, a significant amount of oxygen was found. The Ag was slightly oxidized, but the main component was metallic silver. However, the Ag/Cu sample contained oxygen in an amount far greater than could be attributed to the CuO compound. Assuming the presence of CuCO_3_ and CuO compounds explains the results given in Table 3. In Figure 5, the Cu 2p region of the Ag/Cu sample is presented.

**Figure 6:**
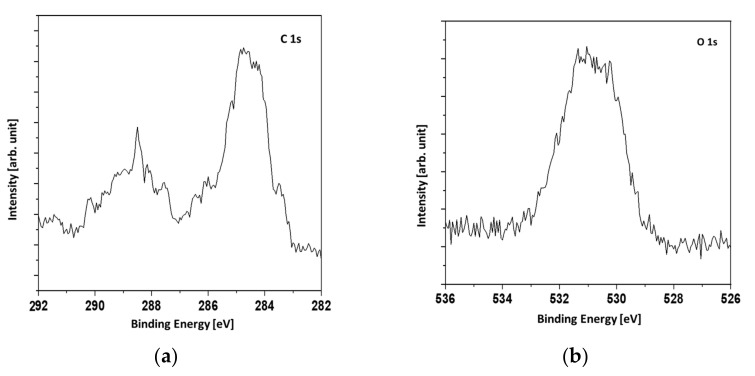
C1s (**a**) and O1s (**b**) high-resolution regions for Ag/Cu sample.

The presence of shakeup signals indicated the presence of copper oxides. The shakeup structure occurs when an ion after ionization by X-rays is left in a higher energy state, a few electron volts above the ground state. The energy to achieve the higher energy state is taken from an emitted photoelectron. Therefore, the shakeups are seen as a signals of higher binding energies than the main lines. The Cu 2p_3/2_ signal was not symmetric with the shoulder evident at higher binding energies. It was concluded that a mixture of metallic copper and copper (II) oxide or a mixture of copper (I) oxide and copper (II) oxide was present. In Figure 6, the C1s and O1s XPS signals are presented. The broadening of the O1s signal denotes two forms of oxygen which can be attributed to copper oxide and the CuCO_3_ compound.

**Figure 7:**
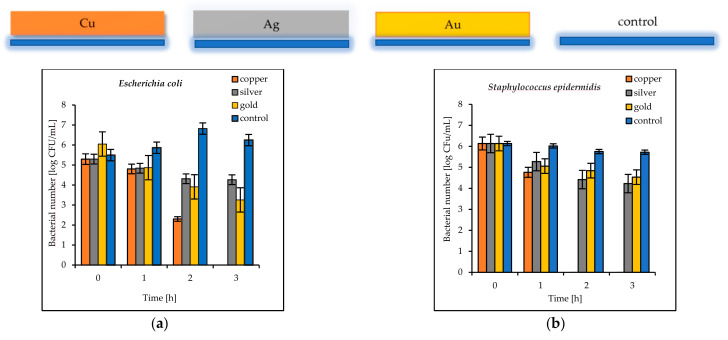
Reduction in bacteria number [log CFU/mL] (**a**) *E. coli*; (**b**) *S. epidermidis* on tested single coatings of copper (Cu), silver (Ag), and gold (Au) and control plates.

The results of the antimicrobial tests illustrated in Figure 7 showed a considerable reduction in the viable Gram-negative and Gram-positive bacteria with increasing exposure time. After 3 h, the log bacterial reduction from the initial “0” time was from 1.21 (on the Ag coatings) to 5.29 (on the Cu coatings) for the *Escherichia coli* bacteria and from 1.60 (on the Au coatings) to 6.12 (on the Cu coatings) for *Staphylococcus epidermidis* (Figure 7a,b). The bacterial sensitivity to the tested metal surfaces was found to vary depending on the microbial species. Based upon the results, the more sensitive species was the Gram-positive *S. epidermidis*.

**Figure 8:**
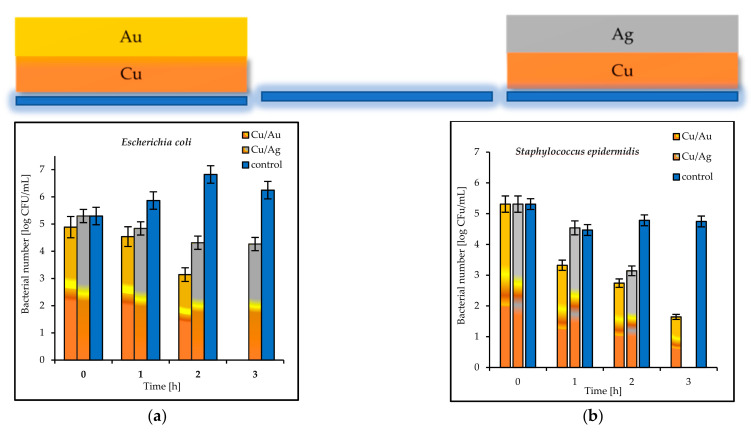
Reduction in bacteria number [log CFU/mL] (**a**) *E. coli*; (**b**) *S. epidermidis* bacteria on tested double coatings of Cu/Ag and Cu/Au and control plates.

**Figure 9:**
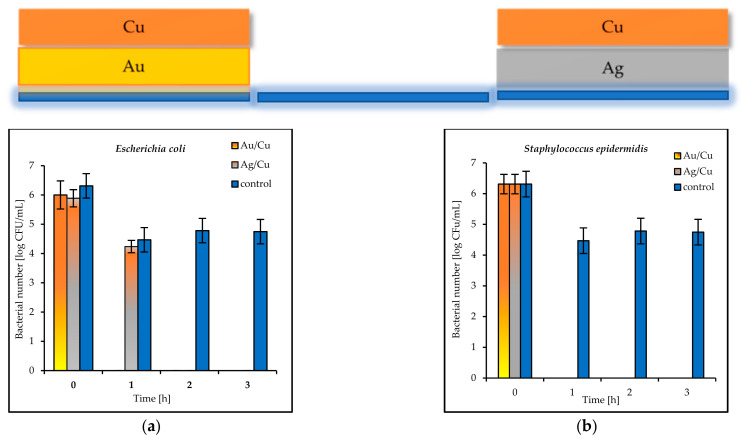
Reduction in bacteria number [log CFU/mL] (**a**) *E. coli*; (**b**) *S. epidermidis* bacteria on tested double coatings of Au/Cu and Ag/Cu and control plates.

The antibacterial properties of the double coatings are presented in Figure 7, Figure 8 and Figure 9.

**Figure 10:**
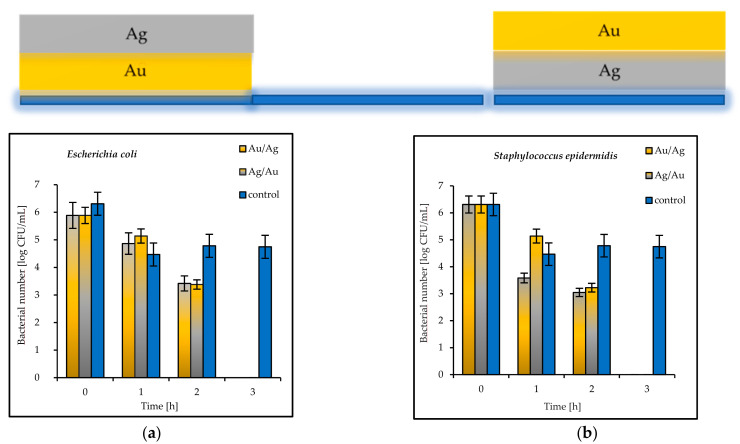
Reduction in bacteria number [log CFU/mL] (**a**) *E. coli*; (**b**) *S. epidermidis* bacteria on tested double coatings of Au/Ag and Ag/Au and control plates.

The tested double coatings consisting of varied combinations of Cu, Ag, and Au layers had antibacterial activity equal to or higher than that of the single coatings. It was shown that when copper was covered by silver (Cu/Ag), the number of *E. coli* bacteria was 6 log after 3 h. Almost the same reduction (5.5 log) in the same time was obtained for *S. epidermidis* on the plates coated with Cu/Ag (Figure 8a,b). As a rule, a reduction of 6 log is equivalent to a 99.9999% reduction. The best antibacterial properties, which indicated a fast reduction in bacteria in a short period of time (even 1 h), were displayed by the Au/Cu and Ag/Cu coatings (Figure 9a,b). As a result, the Ag/Cu coatings achieved better inactivation of *E. coli*, while the Au/Ag coating achieved better inactivation of *S. epidermidis*. The synergistic effect of gold and silver’s bacteriostatic properties was observed for the plates covered with the Ag/Au and Au/Ag layers. After 3 h, total *E. coli* and *S. epidermidis* reduction was observed (Figure 10a,b).

## 4. Discussion

It is well known that copper (Cu), silver (Ag), and gold (Au), in comparison to other noble metals, are considered to have high antimicrobial activity. They have been applied as pure materials or in combination with oxides (e.g., TiO_2_), polymers, chitosan, etc. [7,14,20,23,24,25,26,27]. Attempts to enhance the germ-destroying or bacteriostatic efficacy of surfaces in recent times (since the beginning of the SARS-CoV-2 pandemic) are of great importance. Their application has been mainly focused on reducing pathogenic contamination in public and hospital areas [12,24,25,28]. Because of their smaller size, viruses are more sensitive to commonly used sterilization practices or agents used as disinfectants [10,29]. Nonetheless, the study of viruses is particularly difficult and requires specific biosafety guidelines for their handling (e.g., maintaining an appropriate biosafety level in the laboratory) and poses a danger to laboratory staff. Consequently, starting from the assumption that bacteria and viruses can be spread in similar ways, e.g., by close contact with an infectious person or with infected surfaces and subsequent touching of the mouth, nose, or eyes, and taking into account all available information, it is reasonable to assume that antibacterial surfaces can be antiviral as well. However, there are various methods and in vitro experiments that can be applied to compare fabricated materials and coatings [25]. The choice of standardized test methods is an important step in the development of novel antimicrobial materials. Almost all protocols use model Gram-negative, mainly *Escherichia coli* or *Pseudomonas aeruginosa,* and Gram-positive, e.g., *Staphylococcus aureus,* bacteria [30,31]. In the present study, the ASTM standard was applied to calculate the number of living bacteria. It was proven that this standard was applicable for the fast and reliable testing of single and double coatings of noble metals and for the comparison of their antimicrobial properties.

The maintaining of the excellent antibacterial properties of metallic surface or barrier materials should also be correlated with their mechanical properties such as hardness, anticorrosion performance, and durability. These features can be finetuned by changing the metal ratio in alloys. The most promising compositions are selected based on various factors, such as cost-effectiveness, ease of fabrication, and antibacterial performance. Copper is not a precious metal like gold or silver; thus, it is far less expensive to purchase. 

The magnetron-sputtering (MS) method involves the sputtering of metallic targets and the deposition of nanometric clusters on a substrate located a few centimetres below the target. The method is very useful for incorporating ions on the surface of titanium implants [32,33,34]. The deposition of thin films over metals and alloys is also straightforward. Based on the antimicrobial reduction attained for both strains, and also taking into account the morphological results, it was concluded that the magnetron-sputtering method allowed for the design of novel, multilayer coatings for varied antibacterial applications. Following the deposition of a single copper layer on a glass plate, the viable-cell-count reduction reached a critical rate of 99.9% [13] after ≈180 min (3 h). The same level was obtained in a shorter time of 60 min (1 h) on the surfaces coated with Ag/Cu and Au/Cu in the present study. This means that this material can be considered to be microbiologically clean according to even the strictest US standard, which demands that 99.9% of pathogens must be eliminated within 1 h [30,31].

In a comprehensive review, Villapún et al. summarized that copper elements used in the healthcare sector should be made of mixed metals to optimize the final properties of the resulting alloy [31]. In this study, the significant enhancement of the antibacterial properties of the Cu surfaces, where copper was applied as the outer layer, indicated a bacteria–surface interaction on the magnetron-covered plates. As shown by Müller et al., during wet-plate-testing experiments, Cu surfaces and ion release are the main factors influencing the antibacterial properties as well as the bacterial adhesion, depending on the bacterial hydrophobicity. Gram-negative bacteria have a significantly lower hydrophobicity and zeta potential than Gram-positive bacteria [35,36]. The present study showed that Gram-negative bacteria had lower inactivation rates than Gram-positive bacteria on almost all the tested plates. This may indicate that the main antibacterial factor was the number of cuprous ions present in the suspension. The antibacterial properties increased proportionally to their concentration and absorption on the surface of the bacteria [6,36,37,38]. According to Popov et al., monovalent copper is better for controlling bacterial growth than divalent [39]. According to Nishino et al., metallic Cu did not exhibit antimicrobial activity, whereas Cu^2+^ from CuO was responsible for the antibacterial performance [40]. The main mechanism of ion release involves the hydroxylation of the ions once they are in contact with a wet environment (e.g., the buffers used in this study) or with sweat, physiological fluid, steam from the air, etc. Depending on the media, the formation of HCl, HOCl, or reactive oxygen species (ROS) such as OH, O^2−^, or H_2_O_2_ are observed [31]. The results of the XRD studies indicated that the copper metallic films were characterized by the smallest crystallites (from 3 nm for the Ag/Cu to 5 nm for the Cu coatings). The double-coating systems had smaller crystallinity than the single-coating samples (Cu, Ag, Au). The easily released Cu ions cause the oxidation of the bacterial cell membranes or virus proteins, leading to their death. This rapid process is observed especially in moist environments [35]. As summarized by Vincent et al. [6], the dissolved cuprous ions released from the copper surfaces by the culture medium are responsible for the contact-killing mechanism by membrane damage. It was shown that copper is not genotoxic and does not cause DNA mutations or affect the enzymatic reactions inside the cells. On the other hand, this concept raises a lot of controversy, particularly with regard to photocatalytic materials (e.g., CuO_2_, CuxO@TiO_2_) that present their antimicrobial effect when exposed to light [9,27,33,38]. It is possible that extended ROS generation by induced Fenton-like reactions could lead to an imbalance between the production and accumulation of reactive oxygen species (ROS) in cells (defined as oxidative stress), cell respiration arrest, or even DNA breakdowns. Due to the fact that only small nanoparticles (smaller than ≤20 nm) are able to penetrate inside the cells in this mechanism, the concentration of released ions is probably less important than the size of the NPs. The third proposed mechanism assumes the toxicity of cuprous ions. Cu ions can compete with other metal ions for important protein binding sites or enzymes that cause the inactivation of enzymes and damage to the central catabolic and biosynthetic pathways [37]. As shown by the XPS results, copper was present in native Cu, Cu(I), or Cu(II) oxidative states. 

The differences observed in the antimicrobial ability of Cu, Ag, and Au depended on the varied number of ions released. It could also be assumed that the enhanced antimicrobial activity of the bimetallic hybrids of the Au/Cu and Ag/Cu type was caused by the synergistic effect of the coexistence of both metallic species. Similar results were obtained by Fan et al. [41] and Perdikaki et al. [42]. It was shown that AgCu alloys or Ag/Cu-graphene hybrid samples with varied Ag on stainless steel surfaces exhibited higher antimicrobial activity than other tested samples. Furthermore, the high-power impulse magnetron-sputtering bimetallic Ag/Cu coatings presented higher activity against *Escherichia coli* and *Psuedomonas aeruginosa* than the Ag coating alone. The Ag/Cu coatings deposited at 80, 120, and 160 A, giving Cu/Ag composition ratios of 1.7 to 3.6, showed better inactivation of *E. coli*, whereas those with a lower Cu/Ag ratio gave better inactivation of *P. aeruginosa* [19]. These findings support the results of the present study. It was shown that the lower Cu content (less than 50 ± 3–7 wt.%) in the double coatings Au/Cu and Ag/Cu enhanced the antibacterial performance against *E. coli* and *S. epidermidis.* It was suggested that the better antibacterial properties of the AgCu nanoalloys were caused by the approximately several-dozen-times-greater number of Cu ions released from the nanoalloys than from the single Cu NPs [43]. As shown by Khare et al., the antibacterial effect of bimetallic surfaces consisting of Ag and Cu is long-term [44]. A thorough summary of the microstructures and antimicrobial properties of Ag/Cu combinations was presented by Fan et al. [41]. On the other hand, from the electrochemical point of view, the contact of two metals creates a galvanic pair that facilitates the oxidation of the more active component [45]. The reason for the higher activity of Ag/Cu and Au/Cu was that they work as galvanic couples. Since the electrode potential of copper is less than that of silver and gold, copper is a sacrificial anode (Cu − 2e = Cu^+2^). The copper ion release was improved, which resulted in an improved biocidal effect. We had a natural medium in contact with oxygen, so hydroxyl ions were produced at the cathode site according to the reaction: O_2_ + 2H_2_O + 4 e = 4 OH^−^. Hydroxyl ions are reactive oxygen species, which are able to increase the killing of bacteria. The reason for the high activity of Ag/Cu and Au/Cu could be the combination of two processes: the accelerated release of Cu^+2^ ions due to electrochemical reactions, and the antimicrobial behavior mediated by reactive oxygen species. A similar effect was described by Dowling et al. [46], who observed the enhanced antibacterial effectiveness of Ag-Pt alloy coatings deposited by magnetron sputtering on polymer polyurethane and silicone sheets compared with Ag. They confirmed that the reason for this was the higher release rate of Ag^+^ under the electrochemical action of platinum [46].

The mechanism of the Ag/Cu action deserves further investigation, because the effects may help to optimize the methodology for antibacterial coatings or coatings used to remove microorganisms from indoor aerosols and touch surfaces. 

## 5. Conclusions

Silver, copper, and gold materials were successfully prepared through the magnetron-sputtering method. It was also proven that double coatings containing copper were highly effective against the Gram-negative bacteria *Escherichia coli* and the Gram-positive *Staphylococcus epidermidis*. The antibacterial efficacy of the tested samples followed the order: Ag/Cu > Au/Cu > Cu. Moreover, some insights into the antibacterial strategies or mechanisms of action may enrich our knowledge of metallic surfaces as contact-killing materials for a more effective fight against microorganisms.

## Figures and Tables

**Table 1:** The EDX elemental analysis for single and double coatings.

No	Sample Name	Coating Thickness [nm]	Cu Content [wt.%]	Cu Content Error [wt.%]	Au Content [wt.%]	Au Content Error [wt.%]	Ag Content [wt.%]	Ag Content Error [wt.%]
1.	Cu	100	100					
2.	Au				100			
3.	Ag						100	
4.	Cu/Ag	50/50	56	9			44	7
5.	Cu/Au		47	5	53	3		
6.	Ag/Cu		38	7			62	7
7.	Au/Cu		23	3	77	4		
8.	Ag/Au				33	5	67	7
9.	Au/Ag				73	4	27	4

**Table 2:** The crystallite size on the basis of XRD measurements.

Sample	Mean Crystallite Size [nm]			
	Au	Cu	Ag	Au/Ag
Cu		5		
Ag			50	
Au	56			
Cu/Ag		11	23	
Cu/Au	40	21		
Ag/Cu		3	18	
Au/Cu	34	4		
Ag/Au			10	48
Au/Ag			17	38

**Table 3:** Surface concentration of Ag, Au, and Ag/Cu in %.

Sample	O1	C1s	Cu 2p 3/2	Au	Ag3d
Ag/Cu	54.5	10.5	35.0		
Au				100.0	
Ag	14.3	9.0	-		76.7
